# Highly efficient neutralization of human immunodeficiency viruses by plasma from antiretroviral drug treated patients is mediated by IgG fractions

**DOI:** 10.1186/1742-4690-9-S2-P62

**Published:** 2012-09-13

**Authors:** R Andrabi, M Makhdoomi, R Kumar, M Bala, T Velpandian, K Luthra

**Affiliations:** 1 All India Institute of Medical Sciences (AIIMS), New Delhi, India

## Background

Little is known about the neutralizing activity in patients on antiretroviral therapy (ART), as most recent studies have focused on drug naïve individuals. ART may lead to a significant increase in B cell numbers and normalization of B cell subpopulations, providing a possible explanation for improved B cell responses after ART.

## Methods

Thirty-four HIV-1 seropositive patients on ART (25 males and 9 females) within the age range of 20-55 years were recruited in this study. The patients had a median CD4 count and viral load of 283 cells and 178 RNA copies respectively, and were on treatment for a few days up to two years. Heat inactivated plasma samples were tested for neutralization against a panel of 14 subtype-A, B and C tier 1 and tier 2 viruses in TZM-bl assay.

## Results

Of the 34 plasma samples, remarkably all the plasma samples were able to neutralize at least one virus while 32 (94%) samples were found to neutralize ≥50% viruses tested. Clustering analysis revealed that AIIMS253 (a clade-C virus) was the most sensitive while RHPA4259.7 (a clade-B isolate) was most resistant to antibody neutralization. The Immunoglobulin-G fractions from two representative samples AIIMS221 and AIIMS265 were shown to mediate neutralization exclusively. The IgG fractions retained binding to subtype-A, B and C recombinant gp120 proteins. We did not find any association of mean reciprocal ID50 neutralization titers with the plasma levels of ART drugs and clinical and immunological variables like CD4 count (p=0.35), viral load (p=0.37) and plasma total IgG (p=0.46). However we observed a positive association of neutralization with duration of ART (p=0.02) with a similar trend in two follow up patient samples.

## Conclusion

Plasma antibodies from patients on ART display high neutralizing activity most likely due to an improved B cell function induced by ART despite low antigenic stimulation.

